# Circular RNA hsa_circ_0000515 acts as a miR-326 sponge to promote cervical cancer progression through up-regulation of ELK1

**DOI:** 10.18632/aging.102356

**Published:** 2019-11-26

**Authors:** Qiu Tang, Zhigang Chen, Liangping Zhao

**Affiliations:** 1 Department of Oncology, The Central Hospital of Wuhan, Tongji Medical College, Huazhong University of Science and Technology, Wuhan 430014, China; 2 Department of Gynecology and Obstetrics, Wuhan Central Hospital, Wuhan 430014, China

**Keywords:** cervical cancer, cyclic RNA, Hsa_circ_0000515, microRNA-326, ELK1

## Abstract

This study investigates the role of circular RNA (circRNA) hsa_circ_0000515 in cervical cancer and the underlying mechanism associated with microRNA-326 (miR-326). hsa_circ_0000515 and ETS transcription factor ELK1 (ELK1) were initially over-expressed and miR-326 was down-regulated in cervical cancer tissues and cells. Low hsa_circ_0000515 expression was found to be associated with favorable prognosis of patients with cervical cancer. A series of mimics, inhibitors, over-expression plasmids or siRNAs were introduced into cervical cancer cells to alter the expression of hsa_circ_0000515, miR-326 and ELK1. *In vitro* experiments exhibited that silencing of hsa_circ_0000515 or upregulation of miR-326 resulted in suppressed proliferation and invasion, along with induced apoptosis and autophagy of cervical cancer cells. Dual-luciferase reporter assay, RNA pull-down and RIP assays highlighted that hsa_circ_0000515 was able to act as a ceRNA of miR-326 to increase ELK1. Furthermore, enhancement of ELK1 expression resulted in enhanced proliferation and invasion but repressed apoptosis and autophagy of cervical cancer cells. *In vivo* experiments further confirmed the suppressed tumor growth by hsa_circ_0000515 silencing. Our findings demonstrated that hsa_circ_0000515 acts as a tumor promoter in cervical cancer. The study provides evidence for targeting hsa_circ_0000515 for therapeutic purposes in treating cervical cancer.

## INTRODUCTION

Cervical cancer is the second leading cancer-related cause of death in women aged from 20 to 39 years in 2019 [1]. Hysterectomy has the largest effect on long-term survival and remains the primary treatment choice for early-stage cervical cancers [2]. Although radiotherapy has advancement in therapy-related toxicity for patients with locally-advanced tumors [3], patients with invasive cervical cancer may still suffer from a risk of secondary malignancies following definitive radiotherapy [4]. In the recent years, molecular analyses have shed light on novel therapeutic targets based on comprehensive genomic characterization for cervical cancer [5].

Circular RNAs (circRNAs) are a group of non-coding RNAs generated from precursor mRNA back-splicing of numerous genes in eukaryotes that are expressed in a cell-specific or tissue-specific manner [6]. Strikingly, circRNAs have emerged as crucial mediators that have great implications in a wide variety of human disease and cancers [7]. The potential of oncogenic or tumor suppressive circRNAs that function as biomarkers have recently been identified [8]. Specifically, many circRNAs are found to be differentially expressed in cervical cancer tissues when compared to adjacent normal tissues through microarray analysis [9]. Microarray expression profiles of differentially expressed circRNAs in cervical cancer (GSE102686) showed that hsa_circ_0000515 was more significantly expressed in the tumor tissues relative to in the normal tissues. However, the exact mechanism of the role of hsa_circ_0000515 in cervical cancer is largely unknown. Some circRNAs function as sponges or competing endogenous RNAs (ceRNA) of microRNAs (miRNAs or miRs) to regulate mRNA expression [10, 11]. A previous study proposed that the circRNA-miRNA-mRNA regulatory networks consist of 5 circRNAs, 2 miRNAs and 7 mRNAs in the pathogenesis of cervical cancer [12]. More specifically, the circRNA hsa_circ_101996 is demonstrated as a tumor suppressor in cervical cancer whereby it acts as a sponge of miR-8075 [13].

MiRNAs are short noncoding RNAs with a length of ~21 nucleotides, which have critical impacts on cervical carcinogenesis [14]. For instance, several miRNAs are identified as tumor promoter such as miR-196a [15], miR-221-3p [16], and some miRNAs like miR-214 [17], miR-218 [18] can act as tumor suppressors. More importantly, miR-326 has been found to impede proliferative, migrating and invasive capabilities by targeting ELK1 in cervical cancer [19]. ELK1, a transcription factor that belongs to the ETS family and ternary complex factor (TCF) subfamily, is found to play key roles in the regulation of cellular growth, differentiation, and survival [20]. Inhibition of ELK1 inhibited cell cycle entry and promoted apoptosis in cervical cancer [21].

Based on those findings, our investigation focuses on the implications and roles of hsa_circ_0000515 in cervical cancer. We aim to shed light on the underlying molecular mechanism that is associated with miR-326 and ELK1. The study demonstrated the hsa_circ_0000515/miR-326/ELK1 network in the carcinogenesis of cervical cancer, which highlights potential novel targets for cervical cancer treatment.

## RESULTS

### Hsa_circ_0000515 and ELK1 are highly expressed while miR-326 is poorly expressed in cervical cancer

**Figure 1:**
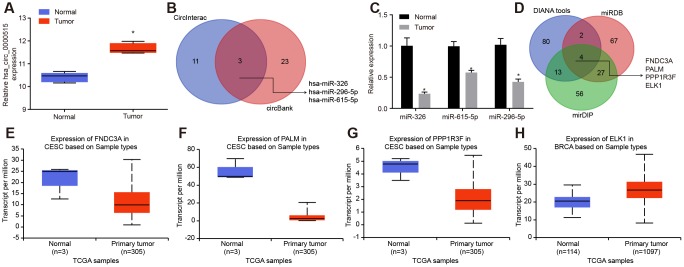
**Bioinformatics prediction of the potential molecules (hsa_circ_0000515, miR-326 and ELK1) that are related to cervical cancer.** (**A**) the expression of hsa_circ_0000515 in the microarray dataset GSE102686; (**B**) the intersected miRNAs might be regulated by hsa_circ_0000515 predicted in CircInteractome and circBank databases; (**C**) the expression of miRNAs in cervical cancer determined by RT-qPCR; (**D**) the Venn diagram of target genes of miR-326 predicted by DIANA tools, miRDB and mirDIP; (**E**–**H**) the expression of FNDC3A, PALM, PPP1R3F and ELK1 in cervical cancer from the UALCAN database.

We initially identified 220 differentially expressed circRNAs in cervical cancer through microarray data analysis (GSE102686). Among them, hsa_circ_0000515 ranked highest with a *p* value of only 3.37E-06. Moreover, limited reports have highlighted the regulatory role of hsa_circ_0000515 in cervical cancer. hsa_circ_0000515 was over-expressed in the cervical cancer samples compared to the paired-paracancerous samples (Figure 1A). Prediction of miRNAs that might be directly regulated by hsa_circ_0000515 was conducted using the CircInteractome and circBank databases. The predicted intersecting miRNAs were miR-326, miR-296-5p and miR-615-5p (Figure 1B). The expression of those miRNAs in cervical cancer tissues was determined by RT-qPCR, among which miR-326 exhibited a lower expression compared to that in the normal adjacent tissues (*p* < 0.05; Figure 1C). We therefore speculated that hsa_circ_0000515 might regulate miR-326 to affect the of progression cervical cancer. Subsequently, the target genes of miR-326 were predicted by DIANA tools, miRDB and mirDIP databases, and the Venn diagram of intersected candidate target genes was plotted. We identified four intersected genes FNDC3A, PALM, PPP1R3F and ELK1 (Figure 1D). The expression of those genes in cervical cancer were analyzed on UALCAN database. Compared with the normal group, ELK1 expression was the highest compared to the rest of the genes, which were mildly elevated in cervical cancer tissues (Figure 1E–1H). Therefore, we speculated that hsa_circ_0000515, miR-326 and ELK1 were involved in cervical cancer.

### Hsa_circ_0000515 is highly expressed in cervical cancer tissues and cells

**Figure 2:**
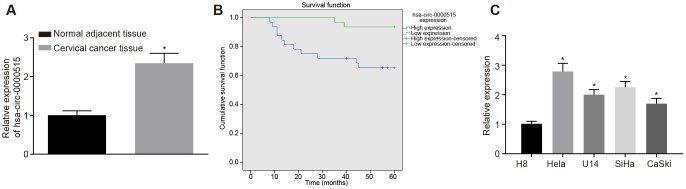
**Hsa_circ_0000515 is expressed at high levels in cervical cancer tissues and cells.** (**A**) the expression of hsa_circ_0000515 in cervical cancer and normal adjacent tissues determined by RT-qPCR; *p < 0.05, compared with normal adjacent tissues; (**B**) overall survival of patients with high expression or low expression of hsa_circ_0000515 with the mean expression of hsa_circ_0000515 as the cut-off value; (**C**) hsa_circ_0000515 expression in normal cervical cell line (H8) and 4 cervical cancer cell lines (Hela, U14, SiHa, CaSki) determined by RT-qPCR; *p < 0.05 *vs.* the normal cervical cell line H8; measurement data were expressed as mean ± standard deviation; unpaired *t* test was used to compare data with normal distribution and equal variance between cervical cancer (n = 63) and normal adjacent tissues (n = 40); one-way ANOVA was applied for comparison of data among multiple groups, followed by Tukey's post hoc test. The survival rate of the patients was calculated by Kaplan-Meier method, and the univariate analysis was performed by Log-rank test. The cell experiments were repeated 3 times independently.

The expression of hsa-circ-0000515 in the cervical cancer tissues and the normal adjacent tissues was determined by RT-qPCR. The results showed that the expression of hsa_circ_0000515 in cervical cancer tissues was significantly higher than that in normal adjacent tissues (*p* < 0.05; Figure 2A). Then, the patients were assigned into two groups, with the mean expression of hsa_circ_0000515 as the cut-off value. The follow-up data showed that patients with low expression of hsa_circ_0000515 had a longer overall survival rate compared to patients with high expression (Figure 2B). The expression of hsa_circ_0000515 in normal cervical cell line (H8) and 4 cervical cancer cell lines (Hela, U14, SiHa, CaSki) was determined. It was found that the expression of hsa_circ_0000515 in all four cervical cancer cell lines were elevated compared with the levels in normal cervical cell line H8 (*p* < 0.05; Figure 2C). Moreover, Hela and SiHa cell lines exhibited the highest expression of hsa_circ_0000515. Therefore, the Hela and SiHa cell lines were selected for subsequent experiments.

### Silencing of hsa_circ_0000515 attenuates proliferation and invasion of cervical cancer cells, and promotes apoptosis and autophagy

**Figure 3:**
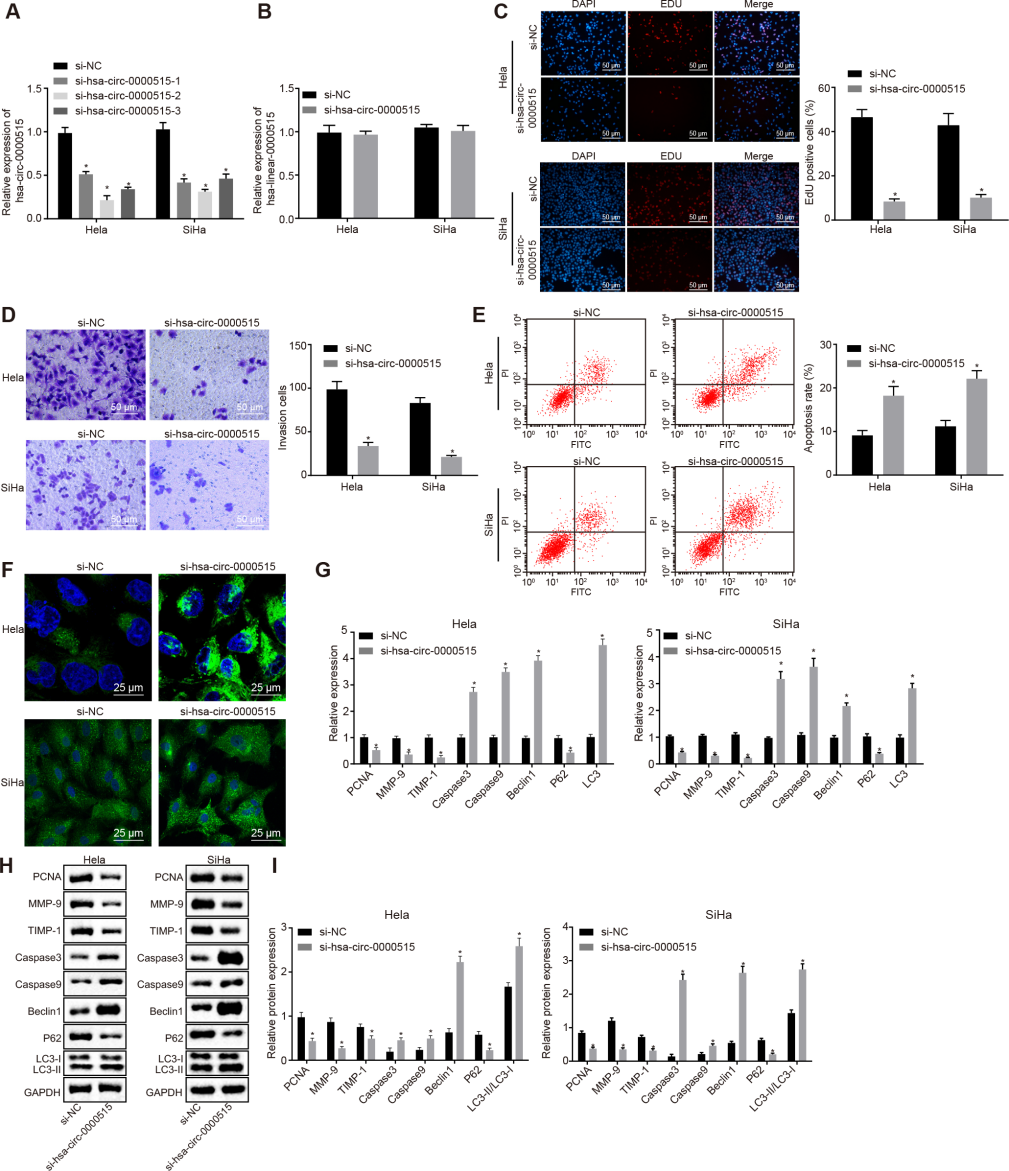
**Silencing of hsa_circ_0000515 attenuates cell proliferation and invasion, and enhances apoptosis and autophagy in Hela and SiHa cells.** (**A**) effect of three different siRNA sequences targeting hsa_circ_0000515 on its expression determined by RT-qPCR; (**B**) expression of hsa_linear_0000515 determined by RT-qPCR; (**C**) EdU-stained cells (200 ×) and proliferation of cervical cancer cells; (**D**) cells invaded through the membrane in a Transwell system (200 ×) and invasion ability of cervical cancer cells; (**E**) flow cytometric data showing cervical cancer cell apoptosis; (**F**) autophagosome formation observed by MDC staining (400 ×) and quantitative analysis of the autophagosome number; (**G**) mRNA and protein expression of proliferation-related gene (PCNA), apoptosis-related genes (Caspase3 and Caspase9), invasion-related genes (MMP-9 and TIMP-1) and autophagy-related genes (Beclin1, P62 and LC3-II/LC3-I) determined by RT-qPCR; (**H** and **I**) cellular protein expression of PCNA, Caspase3, Caspase9, MMP-9, TIMP-1, Beclin1, P62, LC3-I and LC3-II measured by Western blot assay. *p < 0.05 *vs.* the si-NC group (cervical cancer cells transfected with si-NC). Measurement data were expressed as mean ± standard deviation. Unpaired *t* test was used to compare data with normal distribution and equal variance between two groups. The cell experiment is repeated three times independently.

In order to verify the biological functions of hsa_circ_0000515 in cervical cancer cells, three specific siRNA sequences targeting hsa_circ_0000515 (si-hsa_circ_0000515) were designed. RT-qPCR was then used to measure hsa_circ_0000515 expression (Figure 3A). The results suggested that the interference degree of si-hsa_circ_0000515-2 was the most significant. Furthermore, the expression of hsa_circ_0000515 and hsa_linear_0000515 was examined in two cervical cancer cell lines (Hela and SiHa) using RT-qPCR (Figure 3B). The results demonstrated that hsa_circ_0000515 expression was significantly lower in cells treated with si-hsa_circ_0000515, compared to those treated with si-NC. Additionally, there were no significant differences in hsa_linear_0000515 expression in the two cell lines. The aforementioned results suggest that it is hsa_circ_0000515 rather than hsa_linear_0000515 that exerts effects in cervical cancer cells and the expression of hsa_circ_0000515 could be specifically altered. Thus, the si-hsa_circ_0000515-2 was selected to knock down the expression of hsa_circ_0000515 for subsequent assays. To identify whether hsa_circ_0000515 affected proliferation and apoptosis of cervical cancer cell lines (Hela and SiHa), EdU, Transwell, MDC staining and flow cytometry were conducted to assess the role of hsa_circ_0000515 in the two cervical cancer cell lines. EdU test and Transwell assay results showed that the proliferative and invasive abilities of cervical cancer cells transfected with si-hsa_circ_0000515 were significantly reduced (*p* < 0.05; Figure 3C–3D). Flow cytometric and MDC staining data showed that the proportion of apoptotic cells and the number of formed autophagosomes were increased after si-has-circ-0000515 transfection (*p* < 0.05; Figure 3E–3F).

In addition, the mRNA and protein expression of proliferation-related gene PCNA, apoptosis-related genes (Caspase3 and Caspase9), invasion-related genes (MMP-9 and TIMP-1) and autophagy-related genes (Beclin1, P62, LC3-I and LC3-II) was further determined in cervical cancer cell lines (Hela and SiHa) by RT-qPCR and western blot analysis, respectively. The results displayed that the mRNA and protein expression of PCNA, MMP-9, TIMP-1 and P62 was decreased after hsa_circ_0000515 silencing, while the mRNA and protein expression of Caspase3, Caspase9, Beclin1 and LC3, and the ratio of LC3-II/LC3-I was increased (*p* < 0.05; Figure 3G–3I). These results suggested that silencing of hsa_circ_0000515 inhibited proliferation and invasion and promoted apoptosis and autophagy of Hela and SiHa cells.

### Hsa_circ_0000515 functions as a sponge of miR-326

**Figure 4:**
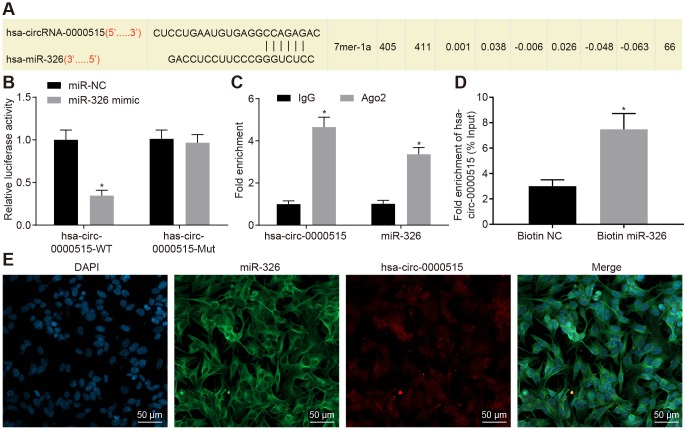
**Hsa_circ_0000515 specifically binds to miR-326.** (**A**) the miR-326 binding site in hsa_circ_0000515 predicted by bioinformatics websites (Starbase V2.0 and Circinteractome); (**B**) the relationship between hsa_circ_0000515 and miR-326 confirmed by dual luciferase reporter assay, *p < 0.05 *vs.* the si-NC group, (**C**) hsa_circ_0000515 and miR-326 co-immunoprecipitated with Ago2 revealed by RIP assay; (**D**) hsa_circ_0000515 pulled-down with biotin-labeled miR-326 by RNA pull-down assay, *p < 0.05 *vs.* the biotin-NC group; (**E**) FISH analysis of the localization of hsa_circ_0000515 and miR-326 in cervical cancer cells (200 ×). *p < 0.05 *vs.* the IgG or biotin-NC group. Measurement data were expressed as mean ± standard deviation. Unpaired *t* test was used to compare data with normal distribution and equal variance between two groups. The cell experiment is repeated three times independently.

It was predicted that hsa_circ_0000515 could bind to miR-326 (Starbase V2.0 and Circinteractome) (Figure 4A). The interaction between hsa_circ_0000515 and miR-326 was verified by the dual luciferase reporter gene assay. The results demonstrated that the luciferase activity of hsa_circ_0000515-wt was inhibited by miR-326 (*p* < 0.05), and the luciferase activity of hsa_circ_0000515-mut was not affected (Figure 4B). This suggested that miR-326 could specifically bind to hsa_circ_0000515. Furthermore, RIP assay showed that the content of Ago2 binding to hsa_circ_0000515 and miR-326 binding was increased, as compared with IgG (*p* < 0.05). This indicated that both hsa_circ_0000515 and miR-326 could bind to Ago2 protein (Figure 4C). RNA pull-down experiments showed a significant increase in hsa_circ_0000515 pulled-down by biotin-labeled miR-326 (*p* < 0.05) (Figure 4D), indicating that hsa_circ_0000515 can directly bind to miR-326. Furthermore, FISH analysis in cervical cancer cells showed that hsa_circ_0000515 and miR-326 were co-localized in the cytoplasm (Figure 4E). Hence, our evidence demonstrated that hsa_circ_0000515 could directly bind to miR-326.

### miR-326 exerts inhibitory effects on cervical cancer cells and reverses the promotive roles of hsa_circ_0000515

**Figure 5:**
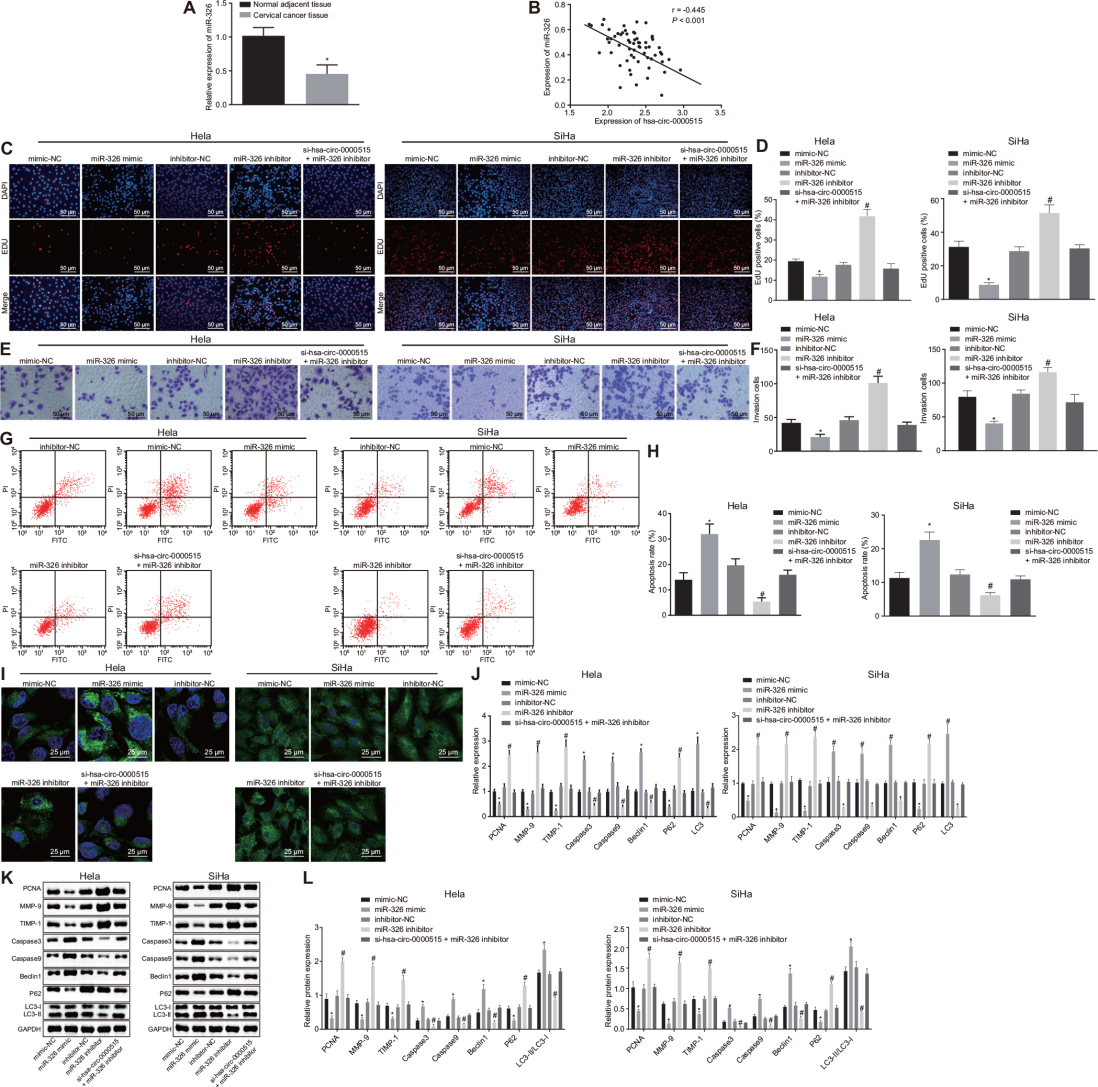
**Hsa_circ_0000515 accelerates cell proliferation, invasion, but attenuates cell apoptosis and autophagy of cervical cancer cells *via* miR-326 binding.** Hela and SiHa cells were transfected with miR-326 mimic or inhibitor alone or co-transfected with si-hsa_circ_0000515. (**A**) the expression of miR-326 relative to U6 determined by RT-qPCR. *p < 0.05 *vs.* normal adjacent tissues; (**B**) Pearson correlation analysis of hsa_circ_0000515 and miR-326 expression; (**C**–**D**) EdU-stained cells (200 ×) and proliferation ability of cervical cancer cells; (**E**–**F**) cells invaded through the membrane in a Transwell system (200 ×) and the number of invaded cervical cancer cells; (**G**–**H**) apoptosis ability evaluated by flow cytometry; (**I**) MDC staining showing autophagosome formation (400 ×) and quantitative analysis of the autophagosome number; (**J**) mRNA expression of PCNA, Caspase3, Caspase9, MMP-9, TIMP-1, Beclin1, P62, LC3-I and LC3-II determined by RT-qPCR; (**K**–**L**) protein expression of PCNA, Caspase3, Caspase9, MMP-9, TIMP-1, Beclin1, P62, LC3-I and LC3-II measured by Western blot assay. *p < 0.05 *vs.* the mimic-NC group; #*p* < 0.05 *vs.* the inhibitor-NC group; measurement data were expressed as mean ± standard deviation; unpaired *t* test was used to compare data with normal distribution and equal variance between cervical cancer (n = 63) and normal adjacent tissues (n = 40); one-way ANOVA was applied for comparison of data among multiple groups, followed by Tukey's post hoc test. The cell experiments repeated 3 times.

The above assays showed that hsa_circ_0000515 could bind to miR-326. Thereafter, it was further analyzed whether miR-326 was involved in the role has-circ-0000515 played in the progression of cervical cancer. The expression of miR-326 in cervical cancer tissues was lower than that in normal adjacent tissues (Figure 5A). Pearson correlation analysis showed a negative correlation between miR-326 expression and hsa_circ_0000515 expression (Figure 5B).

Furthermore, miR-326 expression was altered by mimic or inhibitor in order to determine its role in cervical cancer cells. Besides, miR-326 mimic or inhibitor was subsequently co-transfected with si-hsa_circ_0000515 into Hela cells to analyze the mechanism underlying the oncogenic role of hsa_circ_0000515 associated with miR-326. As seen from the results of EdU and Transwell assays (Figure 5C–5E), the proliferative and invasive capacities of Hela and SiHa cells were inhibited by transfection with miR-326 mimic but enhanced by transfection with miR-326 inhibitor (*p* < 0.05). Proliferative and invasive capacities witnessed no significant change in hsa_circ_0000515-silenced and miR-326-inhibited cervical cancer cells, suggesting that their effects could be antagonized. Meanwhile, flow cytometric data and MDC staining revealed that the apoptosis rate as well as autophagosome formation of Hela cells were significantly increased by enhancement of miR-326 expression (*p* < 0.05), while inhibition of miR-326 reduced the apoptosis rate as well as the number of formed autophagosomes, which was antagonized by silencing of has-circ-0000515 (Figure 5G–5I). The mRNA and protein expression of PCNA, MMP-9, TIMP-1 and P62 was decreased in Hela cells transfected with miR-326 mimic, whereas mRNA and protein expression of Caspase3, Caspase9, Beclin1 and LC3 as well as LC3-II/LC3-I ratio was increased (*p* < 0.05). In contrast, the mRNA and protein expression of PCNA, MMP-9, TIMP-1 and P62 was elevated but Caspase3, Caspase9, Beclin1 and LC3 as well as LC3-II/LC3-I ratio was decreased when miR-326 was suppressed (*p* < 0.05). However, all those were antagonized by silencing of has-circ-0000515. Meanwhile, no significant change was observed in hsa_circ_0000515-silenced and miR-326-inhibited cervical cancer Hela and SiHa cell lines (Figure 5J–5L). These results suggested that miR-326 upregulation led to attenuated proliferation and invasion, and induced apoptosis and autophagy of Hela and SiHa cells, which could be antagonized by hsa_circ_0000515 silencing.

### ELK1, a target gene of miR-326, facilitates proliferation and invasion, and impedes apoptosis and autophagy of Hela and SiHa cells

**Figure 6:**
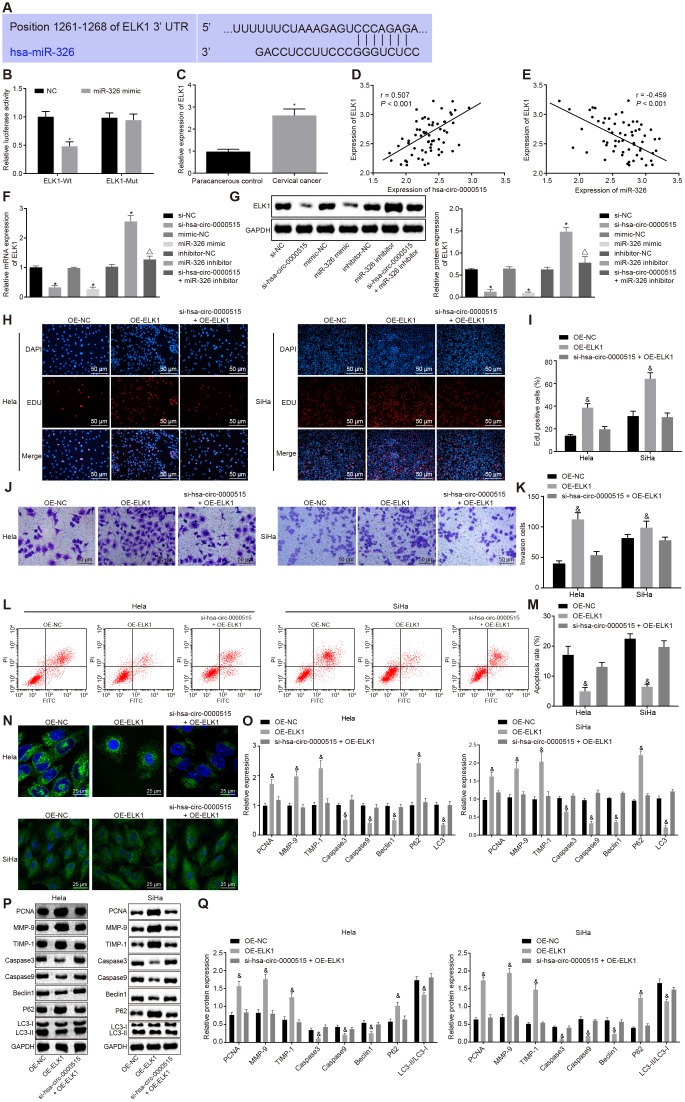
**ELK1 targeted by miR-326 induces proliferation and invasion, and attenuates apoptosis and autophagy of cervical cancer cells.** (**A**) the functional miR-326 binding sites in ELK1 3′UTR; (**B**) the relationship miR-326 and ELK1 analyzed by the dual luciferase reporter assay; (**C**) ELK1 expression in the normal adjacent tissues and the cervical cancer tissues determined by RT-qPCR; (**D**) Pearson correlation analysis of hsa_circ_0000515 and ELK1 expression; (**E**) Pearson correlation analysis of miR-326 and ELK1 expression; (**F**) ELK1 mRNA expression in cervical cancer cells determined by RT-qPCR; (**G**) ELK1 protein expression in cervical cancer cells determined by Western blot assay; (**H**–**I**) EdU-stained cells (200 ×) and proliferation ability of Hela and SiHa cells assessed by EdU assay; (**J**–**K**) cells invaded through the membrane in a Transwell system (200 ×) and the number of invaded Hela and SiHa cells; (**L**–**M**) apoptosis ability evaluated by flow cytometry; (**N**) MDC staining showing autophagosome formation (400 ×) and quantitative analysis of the autophagosome number; (**O**) mRNA expression of PCNA, Caspase3, Caspase9, MMP-9, TIMP-1, Beclin1, P62, LC3-I and LC3-II determined by RT-qPCR; (**Q**) Western blots and protein expression of PCNA, Caspase3, Caspase9, MMP-9, TIMP-1, Beclin1, P62, LC3-I and LC3-II. *p < 0.05 *vs.* the NC group; #*p* < 0.05 *vs.* the normal adjacent group; &*p* < 0.05 *vs.* the OE-NC group; measurement data were expressed as mean ± standard deviation; unpaired *t* test was used to compare data with normal distribution and equal variance between cervical cancer (n = 63) and normal adjacent tissues (n = 40); one-way ANOVA was applied for comparison of data among multiple groups, followed by Tukey's post hoc test. The cell experiments repeated 3 times.

The sequence containing functional miR-326 binding sites in ELK1 3’UTR was initially predicted by bioinformatic analysis (Figure 6A). The dual luciferase reporter gene assay further verified that the luciferase activity of ELK1-wt 3'UTR was inhibited by transfection with miR-326 mimic (*p* < 0.05), while the luciferase activity of ELK1-mut 3'UTR was not inhibited (*p* > 0.05), suggesting that miR-326 could specifically bind to the ELK1 3'UTR (Figure 6B). The expression of ELK1 in cervical cancer tissues was increased when compared to the normal adjacent tissues (*p* < 0.05) (Figure 6C). Pearson correlation analysis showed a positive correlation between the expression of ELK1 and that of hsa_circ_0000515 (Figure 6D), and a negative correlation between the expression of ELK1 and that of miR-326 (Figure 6E). Western blot and RT-qPCR (Figure 6F, 6G) were conducted to measure ELK1 expression in cervical cancer cells, which showed that ELK1 expression was lower in cervical cancer cells transfected with si-hsa_circ_0000515 or miR-326 mimic, versus those with si-NC or mimic NC. Moreover, cervical cancer cells treated with miR-326 inhibitor exhibited increased ELK1 expression relative to those with inhibitor NC, suggesting that miR-326 could repress the expression of ELK1. Meanwhile, ELK1 expression was higher in cervical cancer cells transfected with miR-326 inhibitor in the presence of si-hsa_circ_0000515, relative to those in the absence of si-hsa_circ_0000515, suggesting that hsa_circ_0000515 could upregulate ELK1 expression by repressing miR-326.

To further analyze the role of ELK1 in cervical cancer cells, ELK1 was over-expressed by transfection with OE-ELK1 plasmid in Hela and SiHa cells. The EdU and Transwell assays showed significant increases in the proliferative and invasive abilities of Hela and SiHa cells in response to ELK1 over-expression. On the other hand, no obvious change was observed in Hela and SiHa cells in response to hsa_circ_0000515 silencing and ELK1-over-expression, when compared to those to OE-NC (Figure 6H, 6K). Flow cytometry and MDC staining results highlight that the proportion of apoptotic cells and the number of formed autophagosomes were reduced by treatment of ELK1 over-expression, and no obvious change was observed in cervical cancer cells in the presence of hsa_circ_0000515 silencing and ELK1-over-expression, compared to those of OE-NC (Figure 6L–6N). In addition, the mRNA and protein expression of PCNA, MMP-9, TIMP-1 and P62 (Figure 6O–6Q) was upregulated as a result of ELK1 over-expression, and the mRNA and protein expression of Caspase3, Caspase9, Beclin1 and LC3 as well as the ratio of LC3-II/LC3-I was significantly lowered (*p* < 0.05). No obvious change was observed in cervical cancer cells in response to hsa_circ_0000515 silencing and ELK1-over-expression. These findings demonstrated that ELK1 over-expression also plays an oncogenic role in cervical cancer, which could reverse the effect of hsa_circ_0000515 silencing on cervical cancer cells.

### Silencing of hsa_circ_0000515 inhibits tumor growth *in vivo*

**Figure 7:**
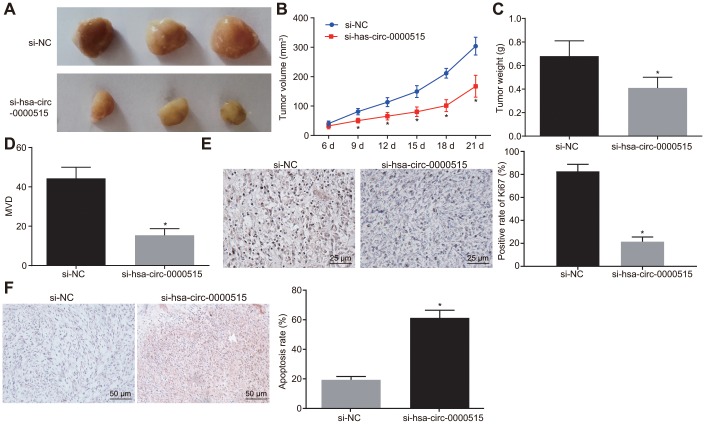
**Hsa_circ_0000515 silencing *in vivo* inhibits tumor growth in nude mice.** (**A**) tumors formed in nude mice at 21^st^ d; (**B**) tumor growth curve of nude mice; (**C**) tumor weight in nude mice at 21^st^ d; (**D**) immunohistochemical detection of MVD density in tumor tissues of nude mice; (**E**) the immunohistochemical detection of Ki67 in tumor tissues of nude mice (400 ×); (**F**) cell apoptosis in tumor tissues assessed by TUNEL staining (200 ×); *p < 0.05 *vs.* the si-NC group; measurement data were expressed as mean ± standard deviation; unpaired *t* test was used to compare data with normal distribution and equal variance between two groups; data at different time points were compared by repeated measures analysis of variance, with Bonferroni post-test; N=10.

The tumor formation experiments in nude mice was conducted to evaluate the effects of hsa_circ_0000515 *in vivo*. Cervical cancer cells stably transduced with sh-hsa_circ_0000515 were injected into nude mice. The results showed that the tumor volume (Figure 7A–7B) and weight (Figure 7C) were reduced by silencing of hsa_circ_0000515 (*p* < 0.05). Immunohistochemical staining produced a significant reduction in MVD density (Figure 7D) and positive Ki67 expression (Figure 7E) in the nude mice tumor tissues in response to silencing of hsa_circ_0000515 (*p* < 0.05). The cell apoptosis in tumor tissues detected by TUNEL staining was markedly increased by inhibition of hsa_circ_0000515 (*p* < 0.05) (Figure 7F). These findings further supported that silencing of hsa_circ_0000515 could inhibit tumor growth *in vivo*.

## DISCUSSION

CircRNAs are newly discovered endogenous non-coding RNAs implicated in cellular processes and transcriptionally or post-transcriptionally modulate gene expression by interacting with miRNAs and other molecules [22]. Our current study focused on the roles of hsa_circ_0000515 in the pathogenesis of cervical cancer, and demonstrated that hsa_circ_0000515 silencing suppressed the proliferative and invasive abilities, and facilitated apoptosis and autophagy of cervical cancer cells. Meanwhile, hsa_circ_0000515 competitively binds to miR-326 to increase ELK1 expression.

We first found that hsa_circ_0000515 was highly expressed in the cervical cancer tissues and cells. Besides, hsa_circ_0000515 served as an oncogenic circRNA and its downregulation led to restrained cell growth and invasive abilities, as well as induced apoptosis and autophagy. In support of our findings, a recent study by Liu et al has also shown that circRNA8924 is highly expressed in cervical cancer tissues and exerts pro-tumor properties [23]. Another circRNA known as circ_0067934 has also been proven to promote tumorigenesis that accelerates proliferation, migration, and invasion of cervical cancer cells [24]. Similarly, circRNA-000284 stimulates proliferation and invasion of cervical cancer cells [25]. The inhibitory effect of hsa_circ_0000515 silencing on cancer progression was observed, as indicated by decreased expression of PCNA, MMP-9, TIMP-1 and P62 in respons to hsa_circ_0000515 silencing, accompanied by elevated expression of Caspase3, Caspase9, Beclin1 and LC3, and the ratio of LC3-II/LC3-I. PCNA is commonly known as a proliferation-related gene. Inhibition of another circRNA CDR1as inhibits the growth of osteosarcoma cells with decreased the expression of PCNA *in vivo* [26]. MMPs such as MMP-9 or their inhibitors like TIMP-1 are associated with oral cancer metastasis and invasion [27]. Similar with our results, the knockdown of lncRNA HAGLROS induced apoptosis along with an increase in the concentration of cleaved-caspase-3, and cleaved-caspase-9 and inhibited autophagy with reductions in the ratio of LC3II/LC3I and Beclin1 expression as well as increase in P62 expression [28]. Furthermore, *in vivo* experiments confirmed that hsa_circ_0000515 hindered tumor growth, which was in compliance with its *in vitro* effects.

Consistent with the low expression levels of miR-326 that determined in the present study, miR-326 was expressed at a low level in primary cervical cancer specimens relative to that in non-cancerous specimens [29]. Furthermore, our data showed that the expression of hsa_circ_0000515 was negatively correlated with miR-326 expression. Meanwhile, our results highly suggest that hsa_circ_0000515 could act as a ceRNA of miR-326 to downregulate its expression. A lot of RNA transcripts including lncRNAs, pseudogenes, and circRNAs could function as ceRNA of miRNAs and have implications in human diseases [30]. LncRNA-HOTAIR enhances cervical cancer cell proliferative and migrating abilities by acting as a sponge of miR-326 [31]. Our study also identified that silencing hsa_circ_0000515 repressed the tumor-promoting effects of down-regulated miR-326 in cervical cancer cells. Specifically, hsa_circ_0000515 acted as a sponge of miR-326 to mediate cervical cancer progression, which is similar with the finding that circPUM1 accelerates the tumorigenesis of lung cancer by acting as a miR-326 sponge [32]. A similar finding demonstrated that hsa_circ_0003998 can also act as a miR-326 sponge which leads to enhanced cell proliferation and invasion in non-small cell lung cancer [33]. Another research suggests that knockdown of hsa_circ_0000263 was able to inhibit cell proliferation and migration ability by controlling the target gene of miR-150-5p [34].

Last but not least, hsa_circ_0000515 acts as a sponge of miR-326 to upregulate ELK1. A more recent study identified miR-326 as a tumor suppressor in human prostatic cancer through inhibiting Mucin1 [35]. ELK1 triggers a proto-oncogene c-Fos, downstream target, by which ELK1 stimulates the growth of bladder cancer cells [36]. In line with our data that ELK1 over-expression promoted cervical cancer development, ELK1 is also able to inhibit the suppressive effects of Tanshinone I on proliferation of HeLa cells [37]. Inhibition of ELK1 is capable of suppressing thyroid cancer progression [38]. Down-regulation of ELK1 is responsible for the oncogenic role of tumor-suppressive miR-135a in breast cancer [39]. The activation of the ERK/ELK1/Snail signaling pathway underlies the promotive function of tumor-derived CXCL5 in colorectal cancer metastasis [40]. The literature mentioned above aids to supports the conclusion that hsa_circ_0000515 silencing could attenuate cervical cancer progression through miR-326-dependent ELK1 inhibition. Interestingly, ELK1 is an integration point associated with different mitogen-activated protein kinase (MAPK) signaling pathways [41]. This therefore gives rise to the highly possible involvement of potential MAPK signaling pathways in the hsa_circ_0000515/miR-326/ELK1 axis a question for the further researches.

**Figure 8:**
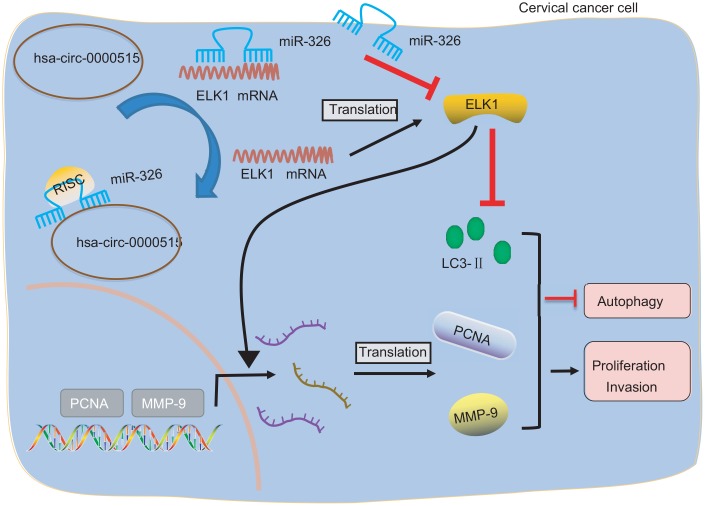
**Image showing the underlying mechanism of how hsa_circ_0000515 is involved in the progression of cervical cancer.** Hsa_circ_0000515 acts as a ceRNA of miR-326 to upregulate ELK1, thereby resulting in promotion of cervical cancer cell proliferation and invasion, and inhibition of apoptosis and autophagy, as reflected by up-regulated PCNA, MMP-9 expression, and down-regulated LC3 expression.

Taken together, our findings demonstrate that hsa_circ_0000515 plays a tumor promotive role in cervical cancer. This study also identified a hsa_circ_0000515/miR-326/ELK1 regulatory, axis where hsa_circ_0000515 diminishes miR-326-dependent inhibition of ELK1 (Figure 8). The findings highlighted the promising uses of hsa_circ_0000515 as a novel target for cervical cancer treatment.

## MATERIALS AND METHODS

### Ethical statement

The procedures carried out in this study have all been approved by the Ethics Committee of Beijing University of Chinese Medicine Third Affiliated Hospital. All patients signed informed consents before their enrollment in the study. Animal experiments were carried out under the approval of the Animal Ethics Committee of Beijing University of Chinese Medicine Third Affiliated Hospital. All the efforts were made to minimize the animal suffering.

### Microarray-based circRNA expression profiling

The cervical cancer-associated circRNA expression profile (GSE102686) was downloaded from the Gene Expression Omnibus (GEO) database (https://www.ncbi.nlm.nih.gov/geo/). R language limma package was used for differential analysis to screen the differentially expressed circRNAs in cervical cancer [42]. After that, potential miRNAs that may bind to the differentially expressed circRNA screened from GSE102686 were predicted by searching the CircInteractome database (https://circinteractome.nia.nih.gov/index.html) and circBank database (http://www.circbank.cn). The candidate miRNAs obtained from the two databases were intersected using Venn (http://bioinformatics.psb.ugent.be/webtools/Venn/). Subsequently, the target genes of miRNAs were predicted by retrieving the mirDIP database (http://ophid.utoronto.ca/mirDIP/), mirDB database (http://www.mirdb.org/) and the DIANA tools (http://diana.imis.athena-innovation.gr/DianaTools/index.php?r=microT_CDS/index). From each database, the top 100 target genes with the highest scores were selected. After selection, the genes were intersected using Venn analysis. The expression of obtained candidate genes in cervical cancer was then identified from the UALCAN database (http://ualcan.path.uab.edu/index.html).

### Study subjects

A total of 63 patients diagnosed with cervical cancer were enrolled in the Beijing University of Chinese Medicine Third Affiliated Hospital. All patients underwent surgical treatment and were confirmed to have cervical cancer by pathological diagnosis. None of the patients received any form of drug treatment prior to surgery and had no signs of distant metastasis. Patients had a mean age of 48.98 ± 9.06 years. Forty cases of normal mucosal tissues 2 cm away from cancer tissue were obtained as normal adjacent tissues. All patients were asked to come in for a follow up period of five years, during which 13 patients had died. The tissues were extracted and frozen at -80°C immediately after the surgery.

### Cell culture and screening

Cervical cancer cell lines (Hela, U14, SiHa, CaSki) and normal cervical epithelial cells H8 were purchased from the Department of Cell Biology, the Institute of Basic Medical Sciences, Chinese Academy of Medical Sciences (Beijing, China). The cells were cultured with RPMI-1640 medium supplemented with 10% fetal bovine serum (FBS), 100 μg/mL streptomycin, and 100 U/mL penicillin in an incubator with 5% CO_2_ at 37°C. Cells were routinely passaged when the cell confluence reached approximately 90%. The medium was discarded, and the cells were washed twice with PBS. After detaching the cells with 0.25% trypsin, the medium was added to terminate trypsinization. Cells were then dissociated into the single cell suspension. Reverse transcription quantitative polymerase chain reaction (RT-qPCR) was performed to determine the expression of hsa_circ_0000515 (http://circrna.org/cgi-bin/simplesearch.cgi) in these cells. The two cell lines with the highest hsa_circ_0000515 expression in cancer cells were selected for subsequent experiments.

### Cell transfection

The cervical cancer cells were plated in a 6-well plate at a density of 3 × 10^5^ cells/well. The cells were transfected with a negative control of siRNA (si-NC), siRNA targeting has-circ-0000515 (si-hsa-circ-0000515), negative control of miR-326 mimic (mimic-NC), miR-326 mimic, miR-326 inhibitor, negative control of miR-326 inhibitor (inhibitor-NC), si-hsa-circ-0000515 + miR-326 inhibitor, negative control of over-expression plasmid (OE-NC), over-expression plasmid of ELK1 (OE-ELK1), or OE-ELK1 + si-hsa-circ-0000515 using lipofectamine 2000 (Invitrogen), when the cell confluence reached 90%. PLenti-GIII-CMV was purchased from Applied Biological Materials (ABM, Canada). The miR-326 mimic, miR-326 inhibitor, si-hsa-circ-0000515, ELK1 OE-ELK1 and their corresponding negative controls (si-NC, mimic-NC, inhibitor-NC and OE-NC) were all purchased from Dharmacon (Lafayette, CO, USA). The sequences of the three siRNAs against hsa_circ_000515 are as follows: “GAGGTGAGTTCCCAGAGAA (siRNA sequence 1), CCGGAGCTTGGAACAGACT (siRNA sequence 2), CCTTTGCCGGAGCTTGGAA (siRNA sequence 3) and the sequence of si-NC is AAGTCGGGTCAAGAGAAGC. The medium was renewed 6 h after transfection. The cells were harvested 36 - 48 h after transfection.

### RNA extraction and quantification

Total RNA was extracted by Trizol (15596-018, Invitrogen, New York, California, USA). The RNA was first reversely transcribed to cDNA according to the instructions provided by the Primescript^TM^ RT reagent (RRO37A, TaKaRa, Dalian, China) with a total system of 25 μL. Reverse transcription of miR-326 and U6 was conducted using the Mir-X™ miRNA First-Strand Synthesis kit (638315, Clontech, USA). The cDNA was subjected to real-time quantitative PCR in accordance with the instructions of the SYBR® Premix Ex Taq^TM^ II Kit (TaKaRa, Dalian, China). PCR was performed in an ABI PRISM^®^ 7300 system (ABI, USA). U6 and GAPDH were used as the internal reference for miR-326 and genes, respectively. All the primers listed in Table 1 were designed and synthesized by Wuhan Bojie Biomedical Science and Technology Co., Ltd (Wuhan, China). The relative expression of each target gene was calculated using the 2^-ΔΔCt^ method [43]. The experiment was repeated three times independently.

**Table 1:** Primer sequences for RT-qPCR.

**Gene**	**Primer sequence**
hsa_circ_0000515	F: 5′- GGTCAGACTGGGCAGGAGAT-3'
	R: 5′- GAGTGACAGGACGCACTCAG -3′
miR-326	F: 5′-CATCTGTCTGTTGGGCTGGA*-3′*
	R: 5′- AGGAAGGGCCCAGAGGCG*-3′*
ELK1	F: 5′- TCCCTGCTTCCTACGCATACA-3′
	R: 5′- GCTGCCACTGGATGGAAACT-3′
MMP-9	F: 5′- TGTACCGCTATGGTTACACTCG-3′
	R: 5′- GGCAGGGACAGTTGCTTCT-3′
TIMP-1	F: 5′- CTTCTGCAATTCCGACCTCGT-3′
	R: 5′- ACGCTGGTATAAGGTGGTCTG-3′
PCNA	F: 5′-GTAGTAAAGATGCCTTCTGGTG-3′
	R: 5′-TCTCTATGGTAACAGCTTCCTC-3′
Caspase3	F: 5′- CATGGAAGCGAATCAATGGACT-3′
	R: 5′- CTGTACCAGACCGAGATGTCA-3′
Beclin1	F: 5′- CCATGCAGGTGAGCTTCGT-3′
	R: 5′- GAATCTGCGAGAGACACCATC-3′
Caspase9	F: 5′- GAACTAACAGGCAAGCAGCAAA-3′
	R: 5′-ATCCTCCAGAACCAATGTCCAC-3′
p62	F: 5′-ACTGATGGCTGTAACGGTCTA-3′
	R: 5′- GGAAGCAGATGGCACAGAGG-3′
LC3	F: 5′-CATGAGCGAGTTGGTCAAGAT*-*3*′*
	R: 5′-TCGTCTTTCTCCTGCTCGTAG*-*3*′*
U6	F: 5′-CTCGCTTCGGCAGCACA-3′
	R: 5′-AACGCTTCACGAATTTGCGT-3′
GAPDH	F: 5′-TGGGTGTGAACCATGAGAAGT-3′
		R: 5′-TGAGTCCTTCCACGATACCAA-3′

Note: RT-qPCR, reverse transcription quantitative polymerase chain reaction; F, forward; R, reverse; miR-326, microRNA-326; ELK1, ETS transcription factor ELK1; MMP-9, matrix metallopeptidase 9; TIMP-1, TIMP metallopeptidase inhibitor 1; PCNA, proliferating cell nuclear antigen; LC3, light chain 3; GAPDH, glyceraldehyde-3-phosphate dehydrogenase.

### Western blot analysis

The total protein in tissues or cells was extracted with radioimmunoprecipitation assay (RIPA) lysis buffer containing phenylmethanesulfonyl fluoride (PMSF). The cell lysate was incubated on ice for 30 minutes and centrifuged at 8000 g for 10 min at 4°C. The total protein was separated by sodium dodecyl sulfate-polyacrylamide gel electrophoresis (SDS-PAGE), and subsequently transferred onto the polyvinylidene difluoride (PVDF) membrane using the wet transfer method. After blocking with 5% skim milk powder for 1 h, the PVDF membrane was then incubated with rabbit antibodies against human ELK1 (ab32106, 1: 500), PCNA (ab152112, 1: 500), Beclin1 (ab207612, 1: 2000), Caspase3 (ab13847, 1: 500), Caspase9 (ab32539, 1: 1000), P62 (ab226386, 1: 2000), LC3-I (ab134178, 1: 1000), or LC3-II (ab48394, 1: 3000) purchased from Abcam at 4°C overnight. On the following day, the membrane was washed three times with tris buffered saline/Tween 20 (TBST), 10 min each time. Horseradish peroxidase (HRP)-labeled goat anti-rabbit immunoglobulin G (IgG) antibody (ab6721, 1: 2000, Abcam) was added as the secondary antibody. The membrane was developed using an enhanced chemiluminescence (ECL) kit (BB-3501, Ameshame, UK). The membrane was photographed by a Bio-Rad image analysis system (Bio-Rad, Hercules, CA, USA) and the band intensities of each protein band were analyzed by Quantity One v4.6.2 software. The experiment was repeated three times.

### 5-Ethynyl-2′-Deoxyuridine (EdU) assay

The cells were incubated with 50 uM EdU solution (EdU solution and cell medium were mixed at a ratio of 1: 1000) for 2 h at room temperature. After fixation with 40 g/L paraformaldehyde for 30 min, the cells were immersed in glycine solution for 8 min. After that, the cells were permeabilized with 0.5% Triton X-100 in PBS. The cells were then stained with Apollo^®^ staining solution at room temperature for 30 min in the dark, and washed twice with methanol and PBS. Finally, Hoechst 33342 reaction solution (C1022, Beyotime Biotechnology Co., Ltd., Shanghai, China) was used for staining at room temperature for 20 min in the dark [44]. Three fields of view were randomly selected, and total number of EdU-stained cells, which indicates proliferating cells and Hoechst 33342-stained cells which indicates the total number of cells, were counted. Cell viability was calculated using the following formula: proliferating cells / total cells × 100%. The experiment was repeated three times.

### Transwell assay

48 hours after transfection, the apical chamber of the Transwell system was coated with Matrigel (50 μL/well; 356234, Becton, Dickinson and Company, USA). The cells were resuspended with serum-free medium to obtain a concentration of 1 × 10^5^ cells/mL and plated in the Matrigel-coated apical chamber. At the same time, 10% FBS medium was added into the basolateral chamber. The Transwell chamber was placed in a 37°C incubator for 24 h. After fixation in 5% glutaraldehyde at 4°C, the cells were stained with 0.1% crystal violet for 30 min, followed by PBS washing. The non-invaded cells were wiped off and the number of cells passing through the Matrigel was counted and recorded. The experiment was repeated three times.

### Flow cytometry

Annexin V-FITC/PI double staining was performed to detect cell apoptosis. Cells were collected 48 h after transfection, and adjusted to a cell concentration of 1 × 10^6^ cells/mL. The cells were then added into a pre-cooled 70% ethanol solution and left overnight at 4°C. Subsequently, 100 μL of cell suspension (not less than 10^6^ cells/mL) were centrifuged and resuspended in 200 μL of binding buffer. The resuspended cells were immediately stained with 10 μL of Annexin V-FITC and 5 μL PI at room temperature for 15 min in the dark. Cell apoptosis was detected on a flow cytometer (Attune NxT, Thermo Fisher, USA) at an excitation wavelength of 488 nm.

### Monodansylcadaverine (MDC) staining

Cell suspension was added to a 6-well plate with 1 × 10^5^ cells/2 mL in each well. Cells were cultured for 24 h at 37°C with 5% CO_2_. The cells were incubated with tinfoil-wrapped MDC (2 μL/well) at 37°C with 5% CO_2_ for 20 min. Cells were then fixed with 4% paraformaldehyde (PFA) at room temperature for 15 min, and washed with PBS followed by treatment with glycerin (glycerin and 10 × PBS at a ratio of 9:1). Finally, MDC staining was observed and photographed under a fluorescence microscope. The autophagosomes were visualized with fluorescence signal distributed in the cytoplasm.

### RNA binding protein immunoprecipitation (RIP)

The cells were lysed using lysis buffer (25 mM Tris-HCl pH 7.4, 150 mM NaCl, 0.5% NP-40, 2 mM EDTA, 1 mM NaF and 0.5 mM dithiothreitol) containing RNasin (Takara) and protease inhibitor (B14001a, Roche, USA). The lysate was centrifuged at 12000 g for 30 min, and the supernatant was incubated with magnetic beads coated with antibody against either Ago-2 (BMFA-1, BioMarker Biotech, China), or anti-IgG in the NC. After incubation at 4 °C for 4 h, the beads were washed three times with the wash buffer (50 mM Tris-HCl, 300 mM NaCl pH 7.4, 1 mM MgCl 2, 0.1% NP-40). RNA was extracted from the magnetic beads using Trizol, and hsa_circ_0000515 expression in which was determined by RT-qPCR.

### RNA pull-down assay

The cells were transfected with 50 nM biotinylated WT-bio-miR-326 or MUT-bio-miR-326. After 48 hours, the cells were harvested and washed with PBS. Cells were lysed in specific lysis buffer (Ambion, Austin, Texas, USA) for 10 min. The lysate was incubated with M-280 streptavidin magnetic beads (S3762, Sigma, USA) pre-coated with RNase-free BSA and yeast tRNA (TRNABAK-RO, Sigma, USA). After incubation at 4°C for 3 h, the beads were washed twice with pre-cooled lysis buffer, thrice with low salt buffer, and once with high salt buffer in successive. The bound RNA was purified by Trizol and then subjected to RT-qPCR to determine hsa_circ_0000515 expression.

### Fluorescence in situ hybridization (FISH)

The subcellular localization of hsa_circ_0000515 and miR-326 was identified by FISH technique according to the instructions provided by the Ribo^TM^ lncRNA FISH Probe Mix (Red) (RiboBio, Guangzhou, China). In detail, the cells were inoculated in a 24-well culture plate at density of 6 × 10^4^ cells/well until 85% confluence. The cells were obtained and fixed with 1 mL of 4% paraformaldehyde at room temperature. After treatment with 2 μg/mL proteinase K, glycine and acetylation reagent, the cells were incubated with 250 μL of pre-hybridization solution overnight. On the following day, cells were washed thrice with PBST, and stained with DAPI (1: 800) for 5 minutes. Following PBST washes, the cells were mounted with anti-fade reagent, observed and photographed under a fluorescence microscope (Olympus, Japan). Five different fields of view were randomly chosen for observation and photographing.

### Dual-luciferase reporter assay

The functional miR-326 binding site in ELK1 3’UTR was predicted using biological prediction website http://www.microrna.org/microrna/home.do. The dual-luciferase reporter assay was conducted to verify whether ELK1 was a direct target of miR-326. The wild type and mutant sequences of ELK1 (hsa_circ_0000515) were constructed under the reporter plasmids M69 pCMV, pGL3 luciferase (Addgene Repository; http://www.addgene.org), and inserted into PGL3 vectors; PGL3-ELK1-wt (PGL3-hsa_circ_0000515-wt) and PGL3-ELK1-mut (PGL3-hsa_circ_0000515-mut). The recombinant plasmids were co-transfected into 293T cells with miR-326 mimic or mimic NC, respectively. Luciferase activity was determined using a Dual-Luciferase^®^ Reporter Assay System (E1910, Promega Corporation, Madison, WI, USA). The relative luciferase activity was expressed by the ratio of firefly luciferase activity to renilla luciferase activity. The experiment was repeated three times.

### Xenograft tumor in nude mice

A total of 20 clean grade female Balb/c nude mice, aged 4-6 weeks and weighing (20 ± 2) g, were purchased from the Animal Experimental Center of Southern Medical University. The cells stably transduced with siRNA targeting hsa_circ_0000515 (si-hsa_circ_0000515) or si-NC were resuspended in 50% Matrigel (BD Biosciences, Bedford, MA). The cell concentration was then adjusted to 2 × 10^6^ cells/mL, respectively. Next, 0.2 mL single cell suspension (containing 4 × 10^5^ cells) was subcutaneously injected into each mouse at the left axilla, with 10 mice in each group. On the 21^th^ day, the tumor weight, volume and lymph node metastasis of the nude mice were assessed after mice were euthanized.

### Immunohistochemistry

The paraffin-embedded tumor tissues were sliced into 5 μm sections and routinely dewaxed. After gradient ethanol dehydration, the sections were washed with PBS thrice, 3 min each time. The sections were immersed in pH of 6.0 buffer, boiled, and then naturally cooled. After rinsing twice with distilled water and PBS, heat antigen retrieval was conducted for 3 min. Afterwards, 1: 9 diluted 30% hydrogen peroxide was added in a dropwise manner to block the endogenous peroxidase activity. Primary anti-human Ki67 (ab92742, 1: 500) or CD34 (ab81289, 1: 2500) was incubated with the sections at room temperature for 2 h. Biotin-labeled secondary antibody to IgG (ab6721, 1: 1000, abcam, USA) was added for 20-min incubation at room temperature. The sections were incubated with the streptomycin peroxidase solution for 10 min at room temperature, and stained with diaminobenzidine (DAB) for 8 min followed by counterstaining with hematoxylin for 3 min. The staining was observed under the microscope. Ki67 positive staining was expressed in the nucleus. CD34 positive staining presented as a yellow or brownish yellow color, which was mainly located in the cell membrane and space. Microvessel density (MVD) calculation was performed according to the method of Weidner N [45]. The experiment was repeated three times.

### TdT-mediated dUTP-biotin nick end-labeling (TUNEL) staining

The tissue sections were prepared and soaked in washing buffer for 24 h. After thorough rinsing in distilled water, the sections were immersed in 70% ethanol. When the sections dried, they were treated with 0.1% polylysine for 5 min and left to stand at room temperature for 24 h. The coverslips were first soaked in 95% hydrochloric acid, and rinsed with distilled water. The sections were attached to polylysine-treated coverslip at 42°C for 24 h. After that, sections were dewaxed by xylene and hydrated with gradient ethanol followed by another wash in distilled water and PBS, and treated with 20 μg/mL proteinase K at 37°C for 15 min. After immersion in PBS for 10 min at room temperature, the sections were blocked with 50 μL of methanol containing 0.3% H_2_O_2_ at room temperature for 10 min. The sections were permeabilized with 0.1% TritonX-100 containing sodium citrate for 2 min. Following PBS washes, the sections were stained with 50 μL of TUNEL reaction mixture at 37°C for 2 h and washed in PBS. Next, 50 μL of converter-POD was added for reaction at 37°C for 30 min. The sections were colored with 50 μL of DAB for 10 min after PBS washing. After staining, the sections were washed with distilled water and then dehydrated with gradient ethanol of 70%, 80%, 95% I, 95% II, absolute ethanol I, absolute ethanol II for 3 min in each concentration. After xylene clearing and mounting, the section staining was observed under an electron microscope.

### Statistical analysis

SPSS version 21.0 statistical software (IBM Corp., Armonk, NY, USA) was used for statistical analysis. The measurement data were expressed as mean ± standard deviation. The paired data conforming to normal distribution and equal variance between two groups were compared by paired *t* test and unpaired data were compared by unpaired *t* test. Data comparisons between multiple groups were performed using one-way analysis of variance (ANOVA) with Tukey's post hoc test. Data at different time points were compared by repeated measures of ANOVA, with Bonferroni post hoc test. Pearson correlation analysis was conducted to analyze the relationship between the two indicators. The survival rate of the patients was calculated by the Kaplan-Meier method, and the single factor analysis was performed by Log-rank test. Values of *p* < 0.05 were considered statistically significant.
