# Correction for: Circular RNA hsa_circ_0000515 acts as a miR-326 sponge to promote cervical cancer progression through up-regulation of ELK1

**DOI:** 10.18632/aging.102853

**Published:** 2020-02-10

**Authors:** Qiu Tang, Zhigang Chen, Liangping Zhao

**Affiliations:** 1 Department of Oncology, The Central Hospital of Wuhan, Tongji Medical College, Huazhong University of Science and Technology, Wuhan 430014, China; 2 Department of Gynecology and Obstetrics, Wuhan Central Hospital, Wuhan 430014, China

**Keywords:** correction

Original article: Aging. (Albany NY) 2019; 11:9982–9999. PMID: 31772143 PMCID: PMC6914414 https://doi.org/10.18632/aging.102356

**This article has been corrected:** The authors requested to add Hai Xu, as a corresponding author to the author’s list. This author was deleted during the proofreading of the article by mistake. This correction does not change the content of the publication.

The correct list of authors and affiliations is provided below:

Qiu Tang^1,*^, Zhigang Chen^1,*^, Liangping Zhao^2^, Hai Xu^3^

^1^Department of Oncology, The Central Hospital of Wuhan, Tongji Medical College, Huazhong University of Science and Technology, Wuhan 430014, China

 ^2^Department of Gynecology and Obstetrics, Wuhan Central Hospital, Wuhan 430014, China

 ^3^Department of Obstetrics and Gynecology, Huangjiahu Hospital, Hubei University of Chinese Medicine, Wuhan 430065, China

*Co-first authors

**Correspondence to:** Hai Xu; **email:** xuhaihjh@126.com

